# Supplementary material for: Curated and harmonised transcriptomics datasets of interstitial lung diseases
Source: Data Brief. 2025 Oct 14;63:112139. doi: 10.1016/j.dib.2025.112139 (PMC12581653; doi:10.1016/j.dib.2025.112139)
Supplement: Supplementary file 1 [file mmc1.zip › Supplementary_material/RNA-seq/fastQC reports/GSE184316/TRIMMED_SRR15926780_fastqc.html]

TRIMMED\_SRR15926780.fastq.gz FastQC Report 

FastQC Report

Sat 3 Dec 2022  
TRIMMED\_SRR15926780.fastq.gz

## Summary

- Basic Statistics
- Per base sequence quality
- Per sequence quality scores
- Per base sequence content
- Per sequence GC content
- Per base N content
- Sequence Length Distribution
- Sequence Duplication Levels
- Overrepresented sequences
- Adapter Content

## Basic Statistics

| Measure | Value |
| --- | --- |
| Filename | TRIMMED\_SRR15926780.fastq.gz |
| File type | Conventional base calls |
| Encoding | Sanger / Illumina 1.9 |
| Total Sequences | 7216382 |
| Sequences flagged as poor quality | 0 |
| Sequence length | 35-371 |
| %GC | 51 |

## Per base sequence quality

## Per sequence quality scores

## Per base sequence content

## Per sequence GC content

## Per base N content

## Sequence Length Distribution

## Sequence Duplication Levels

## Overrepresented sequences

| Sequence | Count | Percentage | Possible Source |
| --- | --- | --- | --- |
| CAGTGGGGCCACGAGCTGAGTGCGTCCTGTCACTCCACTCCCATGTCCCT | 120221 | 1.6659456220582558 | No Hit |
| AGGCTACACACTGAGGACTCTGTTCCTCCCCTTTCCGCCTAGGGGAAAGT | 106366 | 1.4739519055393686 | No Hit |
| GGGAAGCGGGGAATGTCTACGTGCGTATGCACGTGGCACTCTCTGCCCGA | 83831 | 1.1616763081555272 | No Hit |
| CACCTCCCCGAAGCTCAGGGAGAGCCCTGTTAGGGCCGCCTCTGGCCCTA | 59908 | 0.8301666957209306 | No Hit |
| TGATATGGTTCCTGGCAAGCCCATGTGTGTTGAGAGCTTCTCAGACTATC | 36158 | 0.5010544064879049 | No Hit |
| TTACAAAAGCAGAATAAAAGCGAAAAGAAATGAAAATGTTACACTACATT | 35947 | 0.4981305036235609 | No Hit |
| TGATGACACCCACCGCAACTGTCTGTCTCATATCACGAACAGCAAAGCGA | 34766 | 0.4817649619989629 | No Hit |
| CAATCCACTTGATCCCAACTCATCTCTCATTTATTTCGGCTTCTTTTATT | 33730 | 0.46740873750862966 | No Hit |
| CCCTGGCTGCTACAGGAGAATAGCAGACAGGTATAGTTAAGAAACAACTT | 31476 | 0.43617424909047225 | No Hit |
| AGCTGCTAAGTGCTGTGTTGTCGTTCCCCCTGCTTAAAATAAAGTTGTTT | 29573 | 0.4098036938731902 | No Hit |
| GGCATCAAAAGTGATGGACTGCCCATTGCTGGAGAAGACCTTCTCTCCTA | 28674 | 0.3973459276407485 | No Hit |
| TCAGGAGACGCTCGTAGCCCTCGCGCTTCTCCTCGGCCAATTCGCGGAAG | 26481 | 0.3669567381549369 | No Hit |
| TCAGGAGACGCTCGTAGCCCTCGCGCTTCTCCTCGGCCAGTTCGCGGAAG | 25615 | 0.3549562647875348 | No Hit |
| AGACATCAAATCCTGCAGACAAGGGGAGCCCTCAGTCTGCAGGGCTCCAT | 25159 | 0.3486373088342607 | No Hit |
| CAGCAGTTTACACCTGCTAGGGTGTTCAAAGGTCAGTGCTATAGAAATTC | 23986 | 0.3323826260860359 | No Hit |
| AACAAGCACAAAGCCAAGAAAACCAACGATGCCAGATACTGAATTTCTAT | 22532 | 0.3122340253052014 | No Hit |
| TACCACTGCCACCTTCTCCATCGGCTCCACTGGCCTCGTGGTGTATGACT | 20602 | 0.28548932138015976 | No Hit |
| GGCAATGAGCGGTTCCGCTGCCCTGAGGCACTCTTCCAGCCTTCCTTCCT | 20076 | 0.2782003502586199 | No Hit |
| CTTCATGATGTAGCAGCAGGTGCCAGGGGCTGGCTTGTAGGCGATCAGCA | 18942 | 0.2624861045327146 | No Hit |
| GTCCACGTCACACTTCATGATGGAGTTGAAGGTAGTTTCGTGGATGCCAC | 18392 | 0.25486455678205505 | No Hit |
| GAAACCCAGACACATAGCAATTCAGGAAATTTGACTTTCCATTCTCTGCT | 14529 | 0.20133357685333175 | No Hit |
| CAGCATGTTCCCTCTCCTCATGAGATTGGTGAAGAAAGTATTTGGCAAAG | 13956 | 0.1933933098330992 | No Hit |
| CTCTCTTTCTGGCCTGGAGGCTATCCAGCGTACTCCAAAGATTCAGGTTT | 13925 | 0.19296373168715295 | No Hit |
| CGATCAGCACGGGCTCGTACTCTGCCACAAACTGATCACACTGCTTCTGG | 13810 | 0.19137013533928776 | No Hit |
| ACAAGAAGTACATCAAAGATTACATGAAATCAATCAAAGGGAAACTTGAA | 13144 | 0.1821411338812164 | No Hit |
| ACGTTTACCTGTCCATGTCTTACTACTTTGACCGCGATGATGTGGCTTTG | 12797 | 0.17733263011852754 | No Hit |
| CAATGGGGTACTTCAGGGTCAGGATGCCACGCTTGCTCTGGGCCTCGTCG | 12184 | 0.1688380687164288 | No Hit |
| GTCTCAACATCTGTGTTGGGGAGAGTGGAGACAGACTGACGCGAGCAGCC | 11649 | 0.16142438135896908 | No Hit |
| CAGGACCACCAATGTCATAGGGTGCAATATCTACAATAGGTAGTCTCACA | 11235 | 0.15568743450665443 | No Hit |
| GGCAATGAGCGGTTCCGGTGTCCGGAGGCGCTGTTCCAGCCTTCCTTCCT | 10946 | 0.15168265759767152 | No Hit |
| GGAGGATGGTTGCACGAAACACACTGGGGAATGGAGCAAAACAGTCTTTG | 10737 | 0.14878646945242088 | No Hit |
| GTCCACGTCACACTTCATGATGGAGTTGAAGGTGGTCTCGTGGATGCCGC | 10666 | 0.14780259692460848 | No Hit |
| GGAGAAAAACAGCACCAAGCAGGAGATCCTGGCTGCTCTTGAGAAAGGCT | 10617 | 0.14712358630682246 | No Hit |
| GTTCTGCAGCCCCTGTCATAAAAGGTTTTACTCTTTCTGGTCTCTGTTCT | 10437 | 0.14462926158842476 | No Hit |
| CGTGAATACCAAGACCTGCTCAATGTTAAGATGGCCCTTGACATTGAGAT | 10084 | 0.13973761366845602 | No Hit |
| CTGACAGTGTATCTAGCTTTGGAAAACACAGGGGTCTGCCCTGTGAGCTG | 9973 | 0.13819944675877746 | No Hit |
| ATCAAAGAAGAACATGTGATCATCCAGGCCGAGTTCTATCTGAATCCTGA | 9869 | 0.13675828136592547 | No Hit |
| CGAAGCGGTTGTACTTGCGGATGTAGTGCAGATAGTCTCGGCGGATGACA | 9196 | 0.12743227839102753 | No Hit |
| GGCAGACAAACCAGACATGGGGGAAATCGCCAGCTTCGATAAGGCCAAGC | 8859 | 0.12276234822380522 | No Hit |
| GGATCTTGGCTTTCTCTTTCCTCTTCTCCTCCAGGGTGGCTGTCACTGCC | 8764 | 0.12144589906687311 | No Hit |
| CCAAGATCTTCTTGATGTCCTTGGCGCTGGGGGAGGAGTTGCCCCCTAGG | 8653 | 0.11990773215719455 | No Hit |
| GCGTCTCTGTCTTCTTCAGTTTCGACTTATCGAATTTCTCGATCTCAGCC | 8474 | 0.11742726479834355 | No Hit |
| CACCAAAGTGCGAAAAGTTACTTTCAATGGACTGAACCAGATGATTGTCA | 8451 | 0.11710854552877052 | No Hit |
| TCTTGGGTTATAATCATTGGCAATGTTGTAATCAACAACCCATGTTATTT | 8364 | 0.11590295524821165 | No Hit |
| GGAAAGGGGGCACTGACTGGTGATGCTGATATTGGAGTGCATCTGAGGCT | 8269 | 0.11458650609127954 | No Hit |
| GTGTCCATTCGAGGGCGGATCCTCTCTGGCGTGGTGACCAAGATGAAGAT | 8100 | 0.11224461232789507 | No Hit |
| CAGGGAGGAAAAGTTTGGAAGAGGCAGAGAAATCCTGCTCTCCTCGCCTT | 8021 | 0.11114988092370942 | No Hit |
| TTGGAGTGGGAAGCGGGGAATGTCTACGTGCGTATGCACGTGGCACTCTC | 7995 | 0.11078958957549641 | No Hit |
| CAACTCCTTCATGGTCTCGTCCATCAGCGCCCTCAGTTCCTGGGTGACCT | 7436 | 0.10304332558891699 | No Hit |
| TCGCTCGTACTCGTGCGCCTCGCTTCGCTTTTCCTCCGCAACCATGTCTG | 7405 | 0.10261374744297072 | No Hit |
| CAGTGGGCCACGAGCTGAGTGCGTCCTGTCACTCCACTCCCATGTCCCTT | 7393 | 0.1024474591284109 | No Hit |

## Adapter Content

Produced by FastQC (version 0.11.7)
